# Can *Artemisia herba-alba* Be Useful for Managing COVID-19 and Comorbidities?

**DOI:** 10.3390/molecules27020492

**Published:** 2022-01-13

**Authors:** Anamul Hasan, Partha Biswas, Tohmina Afroze Bondhon, Khoshnur Jannat, Tridib K. Paul, Alok K. Paul, Rownak Jahan, Veeranoot Nissapatorn, Tooba Mahboob, Polrat Wilairatana, Md Nazmul Hasan, Maria de Lourdes Pereira, Christophe Wiart, Mohammed Rahmatullah

**Affiliations:** 1 Department of Biotechnology & Genetic Engineering, University of Development Alternative, Lalmatia, Dhaka 1207, Bangladesh; anamulhasanoris@gmail.com (A.H.); afrozebondhon@gmail.com (T.A.B.); jannat.koli.22@gmail.com (K.J.); paul.kumarov@gmail.com (T.K.P.); rownak86@hotmail.com (R.J.); 2 Laboratory of Pharmaceutical Biotechnology and Bioinformatics, Department of Genetic Engineering and Biotechnology, Jashore University of Science and Technology, Jashore 7408, Bangladesh; partha_160626@just.edu.bd (P.B.); mnhasan1978@gmail.com (M.N.H.); 3 School of Pharmacy and Pharmacology, University of Tasmania, Hobart, TAS 7001, Australia; alok.paul@utas.edu.au; 4 School of Allied Health Sciences, World Union for Herbal Drug Discovery (WUHeDD) and Research Excellence Center for Innovation and Health Products (RECIHP), Walailak University, Nakhon Si Thammarat 80160, Thailand; nissapat@gmail.com (V.N.); tooba666@hotmail.com (T.M.); 5 Department of Clinical Tropical Medicine, Faculty of Tropical Medicine, Mahidol University, Bangkok 10400, Thailand; 6 CICECO-Aveiro Institute of Materials and Department of Medical Sciences, University of Aveiro, 3810-193 Aveiro, Portugal; mlourdespereira@ua.pt; 7 The Institute for Tropical Biology and Conservation, Universiti Malaysia Sabah, Jalan UMS, Kota Kinabalu 88400, Sabah, Malaysia; Christophe.wiart@nottingham.edu.my

**Keywords:** COVID-19, comorbidity, medicinal plants, *Artemisia herba-alba*, molecular docking

## Abstract

The focus of this roadmap is to evaluate the possible efficacy of *Artemisia herba-alba* Asso. (Asteraceae) for the treatment of COVID-19 and some of its symptoms and several comorbidities using a combination of in silico (molecular docking) studies, reported ethnic uses, and pharmacological activity studies of this plant. In this exploratory study, we show that various phytochemicals from *Artemisia herba-alba* can be useful against COVID-19 (in silico studies) and for its associated comorbidities. COVID-19 is a new disease, so reports of any therapeutic treatments against it (traditional or conventional) are scanty. On the other hand, we demonstrate, using *Artemisia herba-alba* as an example, that through a proper search and identification of medicinal plant(s) and their phytochemicals identification using secondary data (published reports) on the plant’s ethnic uses, phytochemical constituents, and pharmacological activities against COVID-19 comorbidities and symptoms coupled with the use of primary data obtained from in silico (molecular docking and molecular dynamics) studies on the binding of the selected plant’s phytochemicals (such as: rutin, 4,5-di-*O*-caffeoylquinic acid, and schaftoside) with various vital components of SARS-CoV-2, it may be possible to rapidly identify plants that are suitable for further research regarding therapeutic use against COVID-19 and its associated symptoms and comorbidities.

## 1. Introduction

In the last days of December 2019, a new viral disease emerged in Wuhan, China [1]. The disease, which was named COVID-19, was found to be caused by a new zoonotic coronavirus of the type of Severe Acute Respiratory Syndrome (SARS) virus, which had previously emerged earlier in mid-November 2002, also in China [2]. The new 2019 virus was designated as SARS-CoV-2. COVID-19 spread quickly, and within months, the World Health Organization had to declare it as a pandemic. As of 16 November 2021, COVID-19, the disease caused by the coronavirus SARS-CoV-2, has affected 224 countries and has caused 254,547,430 infections, resulting in 5,121,625 deaths as well as disruptions to the global economy in magnitudes that have not been seen for more than a hundred years. Despite intensive efforts by scientists, a proper anti-COVID-19 drug has yet to be discovered. However, after almost a year of the viral emergence, several vaccines developed by different companies have obtained ‘emergency’ approvals for administration.

Most of these vaccines need two doses to be administered to obtain maximum efficiency. Considering that the world population is nearing 8 billion [3], this calls for the administration of 16 billion doses or more of vaccine(s), which must be developed while considering possible spoilage and other factors. These are staggering figures, and it is very much possible that vaccines may take more time to reach the populations of low-income countries (LICs) and low-middle-income countries (LMICs), especially when considering the costs involved [4]. A number of questions also remain about the vaccines, such as ‘will vaccine stimulate an immune response, will vaccine provide sustainable immune endurance, will SARS-CoV-2 mutate’ [5], and last but not least, it has to be remembered that these vaccines are directed against COVID-19 only and not against any comorbidities. A comorbidity, which refers to i an already present disease in the patient or an opportunistic serious secondary condition (such as pathogenic infections) is a matter of concern to any patient undergoing surgery or, say, cancer treatment. This is also true for COVID-19 patients, whose prognosis becomes worse if a comorbidity before or after COVID-19 infection is present.

The basic symptoms of COVID-19 are fever, cough, and shortness of breath; however, the disease can rapidly progress to pneumonia, severe acute respiratory distress syndrome (SARDS), multi-organ failure, and death. Older adults with diabetes, hypertension, lung, liver and kidney disease, cancer patients undergoing chemotherapy, or patients taking steroids are more vulnerable to the SARS-CoV-2 virus [6]. In an analysis of 27 papers and 22,753 patient cases of COVID-19, it was found that 27.4% had hypertension, 17.4% had diabetes, and 8.9% had cardiovascular disease (CVD). The comorbidities varied with region, with China having more cases of hypertension (HTN), South Korea having more cases of CVD, and Iran having more cases diabetes than other comorbidities [7]. Together, various African countries account for more than 90% of global malaria cases [8], and a syndromic of COVID-19 and malaria can be a severe challenge from the point of view of diagnosis and treatment [9]. Some worrisome comorbidities that have proven to be fatal in COVID-19 infections, particularly for older adults, are diabetes, hypertension, lung, liver and kidney disease, cancer, and steroid medication [6]. Malaria can be added to this list for COVID-19 patients in India, and tuberculosis, acquired immune deficiency syndrome (AIDS), and malaria can be added to the list of common comorbidities in several sub-Saharan African countries, including Nigeria, the Democratic Republic of Congo, Uganda, Mozambique, and Niger [8,9]. Incidentally, total COVID-19 infection cases in these countries are 1000, 3224, 2661, 4666, and 264, respectively, as of 16 November 2021 (per million population) versus the world average of 32,656 total cases per million population; as of 16 November 2021, the total number of cases on the African continent was 8,638,750 [https://www.worldometers.info/coronavirus/ accessed on: 16 November 2021]. Although the number of COVID-19 cases in sub-Saharan Africa appears to be small, it also should be taken into account that most of these countries are low-income countries (LICs) with poorly developed medical infrastructure. This may make coping with any future surges of COVID-19 with a new SARS-CoV-2 viral variant difficult, as evidenced by recent surges in COVID-19 cases in South Africa with the latest SARS-CoV-2 variant of concern, the omicron mutant [10].

A further problem that researchers and healthcare professionals must face until COVID-19 has been eradicated is how to solve the problem of comorbidities in COVID-19 patients since a large percentage of deaths can be avoided if comorbidities can be treated and/or kept under control. Vaccinations and anti-comorbidity drug(s) are not an answer and do not represent a solution based on recent news reports that vaccines may take some time to reach LICs and LMICs, with richer countries buying and stockpiling most of the vaccines that are manufactured [11].

At the same time, doubts are being expressed by people (and some professionals also) as to whether the vaccines can be totally effective against the emerging variants; notably, the latest variant of concern, omicron or B.1.1.529, is considered as possibly being more resistant to existing vaccines because of the high number of mutations in the spike protein region [12]. A number of investigational and repurposed drugs such as tocilizumab, corticosteroids, lopinavir, ritonavir, and remdesivir, as well as integrated approaches including antivirals and Traditional Chinese Medicine (TCM) treatments are under clinical trials for COVID-19 but have yet to obtain approval [13,14].

Herbal medications may prove to be the way to solve the twin COVID-19 and comorbidity problem, especially in LMICs. The difficulty is that SARS-CoV-2 is a new virus, and so traditional herbal medications have not been evaluated for the treatment of disease caused by this virus. However, traditional medicines are available for the treatment of COVID-19 symptoms and comorbidities since they have been known and treated with herbal medications before. The positive thing regarding this plant-based approach is that effective traditional herbal medications do exist against COVID-19 symptoms and associated comorbidities in traditional African, Indian, and Chinese medicines, and quite a few of these medications have obtained approval for their use by respective authorities in India and China. The World Health Organization has even put out a booklet on traditional medications to be used against fever, common cold, cough, diarrhea, and headache [15]—all of which are symptoms of COVID-19 but vary from individual to individual. It is a fact that many allopathic drugs have been derived from plants and from close observations of traditional medicinal practices. The modern antimalarial drug artemisinin was discovered from *Artemisia annua* following an extensive search of Traditional Chinese Medicine (TCM) treatises [16]. The anti-hypertensive drug reserpine was discovered from an Indian traditional medicinal plant, *Rauwolfia serpentina* [17]. Phytochemicals from various plants are being studied extensively against various SARS-CoV-2 targets in silico; a number of compounds have already been found to have the potential to inhibit the virus through binding with the 3C-like protease or spike protein of CoV-2 [18,19]. 

There are two types of objections against herbal medications—clinical trials on these medications have rarely been done, if any, and a number of these herbal medications have undetermined toxicity. Both of these types of objections can be rapidly answered following rapid clinical trials and toxicity assessments. It is noteworthy that several of the antivirals that are currently under trial also have adverse effects. One of the most promising antivirals, remdesivir (approved by the US FDA or the United States Food and Drug Administration), is contraindicated in patients with renal impairment and carries increased risks of transaminase elevation (according to US FDA). Remdesivir also carries a significant risk of bradycardia [20]. At least thus far, neither vaccines nor conventional drugs (allopathic drugs) have proved to be a panacea for COVID-19.

The real challenge for these types of ‘medicinal plants–pandemic solving approach’ is to search out ‘safe’ plants (without any toxic effects determined by the absence of toxicity reports in traditional uses), whose various phytochemicals can act as possible therapeutics against COVID-19 (initially tested in silico and later to be tested in virucidal studies/clinical trials) and that simultaneously have ethnic uses and pharmacological activity against COVID-19 symptoms and comorbidities or possibly COVID-19 itself. We demonstrate our point with a well-known African plant, *Artemisia herba-alba* Asso. (Asteraceae), which has the added advantage of containing artemisinin, meaning that it can prove to be beneficial in cases or countries where malaria is a major comorbidity. One other objective of this manuscript is to stir up interest in the scientific community, particularly in those who have the necessary bio-safety lab to work with SARS-CoV-2, to try this approach to alleviate COVID-19 and its comorbidities.

## 2. Methods

Secondary data (ethnic uses, pharmacological activity, and phytochemicals) were obtained from published reports in peer-reviewed journals as mentioned in PubMed. The initial search was made using the term <COVID-19 symptoms, medicinal plants>, which yielded 1036 hits. A subsequent search with <COVID-19 symptoms, malaria, medicinal plants> yielded 279 hits. Narrowing down the search with <COVID-19 symptoms, malaria, diabetes, and medicinal plants> reduced the number of hits to 137. Since diabetes and malaria are common in parts of South Asia and most of Africa, and since *Artemisia annua* is the source of the current antimalarial drug, artemisinin, we searched for whether *Artemisia annua* has anti-diabetic uses. As such, we next used the search engine to search for <*Artemisia*, diabetes>, which yielded 2300 hits, but the first hit was on anti-diabetic plants used in the Eastern Cape, South Africa, and contained the plant name of *Artemisia afra* Jacq. (Asteraceae) [21]. Concentrating on the *Artemisia* genera, we hit upon *Artemisia herba-alba*, which reportedly has ethnic usage against diabetes and hypertension in a large section of North African countries [22].

We finally selected *Artemisia herba-alba* for two reasons; phytochemical constituents of the plant have been reported, and the fresh leaves of the plant are used in Northern Africa against the fungus *Mucor rouxii*. This fungus is one of the causative agents of mucormycosis, an often-fatal opportunistic fungal disease in immunocompromised patients, such as COVID-19 patients with diabetes [23,24], and against which any total cure is not only not available, but where the fungus-affected areas may need excision surgery not only of the affected areas but also large portions of adjoining areas due to the contagiousness and fast-spreading rate of the fungus. The fatality rate in cases of disseminated mucormycosis is 70–90%; amphotericin B and isavuconazole are considered first-line therapies against mucormycosis, with posaconazole being used as salvation therapy, but the treatment efficacy of these agents is not yet clear [25]. Interestingly, one of the ethnic uses of the plant *Artemisia herba-alba* is against *Mucor rouxii* infections; the bioactive phytochemicals have also been identified as carvone and piperitone [22]. This is an important issue for the growing number of cases of mucormycosis, particularly in India, as post-comorbidities of mucormycosis in COVID-19 patients necessitates the immediate discovery of suitable measures for patients who are cured of COVID-19 who then die from mucormycosis.

Literature searches of ethnic uses and pharmacological activities of *Artemisia herba-alba* were carried out using PubMed as the major source. The various search terms (keywords) used included <*Artemisia herba-alba* with ethnomedicine or review or pharmacological activities or phytochemicals or indigenous uses> or a combination of more than two terms in the above section. Boolean operators were used with the search terms [26]. It should be clarified in this section is that we did not view any ethnomedicinal uses or pharmacological activity reports as the final say but treated them as supporting or non-supporting documents to build on our hypothesis that *Artemisia herba-alba* may be a suitable plant for further analysis regarding the therapeutic potential of its phytochemicals for the treatment of COVID-19, its symptoms, and/or its comorbidities. It is also emphasized that the final paper selections were based on intense scrutinizing and detailed conversations between the authors involving literally several hundreds of papers in PubMed.

In silico studies (molecular docking) were conducted with various phytochemicals reported to be present in *Artemisia herba-alba* and the 3C-like protease of SARS-CoV-2, also known as the main protease or Mpro [27]. The pdb file (6LU7) of Mpro of SARS-CoV-2 3C-like protease was known before [28] and has been used in the present study. The pdb file contained inhibitor N3 bound Mpro; we conducted molecular docking studies with N3 removed from Mpro. A total of 24 phytochemicals present in *Artemisia herba-alba* [29,30,31] were studied for their binding affinity to Mpro. Ligand molecules were downloaded from Pubchem [32] in sdf format and were optimized with the force field type MMFF94 using Openbable software and were saved as pdbqt format.

Blind molecular docking using AutoDock Vina was carried out for ligand binding [33]. The grid box was generated, aiming to cover the whole main protease, where the center was at X: −26.73, Y: 18.43, Z: 32.37, and the dimensions of the grid box were, X: 80.66, Y: 92.21, and Z: 128.11 (unit of the dimensions, Å). We used exhaustiveness ‘16’ for better ligand and protein binding. The AutoDockVina tool provides a total of nine docked poses for each ligand; among them, pose1 is the best pose because it has the highest binding affinity. We saved pose1 in pdb format using Pymol for further analysis. The predicted binding affinity values were obtained from an average of values from five independent runs of the docking program. The various figures depict the docked pose of the phytochemicals bound to the SARS-CoV-2 main protease (Mpro) as obtained from PyMOL and displayed in Discovery Studio [34].

The 100 ns molecular dynamic (MD) simulations analysed the protein–ligand complex structures to determine the binding consistency of the selected three candidate ligand compounds 4,5-di-*O*-Caffeoylquinic acid (CID-6474309), Rutin (CID-5280805), and Schaftoside (CID-442658) to the targeted protein SARS-CoV-2 Mpro (PDB ID: 6LU7) [35]. The molecular dynamic simulation of the protein–ligand complex structures was carried out using the ‘Desmond v3.6 Program’ in Schrödinger (https://www.schrodinger.com/ accessed on: 16 November 2021) (Paid version) in a Linux environment to analyze the thermodynamic stability of the receptor–ligand complexes [36]. For this framework, a pre-determined TIP3P water approach was formulated to maintain a particular volume with the orthorhombic periodic bounding box shape with a distance of 10 Å. Appropriate ions such as 0+ and 0.15 M salt have been chosen to electrically neutralize the framework and were randomly placed inside the solvent system. Following the construction of the solvency protein system with a ligand complex, the system framework was lowered and relaxed by employing the default protocol implemented by using force field parameters OPLS3e only within Desmond module and the standard protocols [35]. NPT assemblies that made use of the temperature combination of Nose–Hoover and the isotropic approach were kept at 300 K and one atmosphere pressure (101,325 bar) and were preceded by 50 PS capture periods with a total energy of 1.2 kcal/mole. 

All molecular dynamic simulation screenshots were constructed via the maestro application v9.5 of Schrödinger. The simulation event was analyzed using the Simulations Interaction Diagram (SID) of the Desmond modules in the Schrödinger suite, which was used to verify the quality of the MD simulation. The sustainability of the protein–ligand complex structure was assessed by root mean square deviation (RMSD), root mean square fluctuation (RMSF), intramolecular hydrogen bonds, solvent-accessible surface area (SASA) value, protein–ligand contacts (P-L), the radius of gyration (Rg) value, MolSA, and the polar surface area (PSA) according to the trajectory performance. 

In the MD simulations, the root means square deviation (RMSD) was the average distance generated by the dispossession of a single atom over a specified period compared to a reference time [36]. The root mean square deviation (RMSD) of protein structural atoms such as Cα, backbone, sidechain, and heavier particles was calculated first, followed by the RMSD of the protein fit ligand atoms from all time frames, which are aligned and assessed against the reference time (in our study 100 ns). The RMSD of an MD simulation with a period of x can be determined using the equation below (Equation (1)).

(1) $$\mathrm{RMSDx}=\sqrt{\frac{1}{N}}\sum_{i=1}^{N}(r^{\prime }i(t_{x}))-r_{i}\;(t_{\mathrm{ref}}){)}^{2}$$

Here, N represents the number of atoms chosen; tref is the reference time, and r’ conveys the placement of the bit selected in the system x after superimposing the point of the reference system. 

The root mean square fluctuation (RMSF) has been primarily used to identify and monitor local changes in the conformational structure within the protein complex [37]. The continuity equation (Equation (2)) can be used to determine the RMSF value of an MD simulation of a protein with the number of residues 2.

(2) $$\mathrm{RMSFi}=\sqrt{\frac{1}{T}}\;\sum_{t=1}^{T}<(r^{\prime }i\;(t))-r_{i}\;(t_{\mathrm{ref}}))2^{\;}>$$

Here, T generally denotes the trajectory time; tref denotes the reference or given time; r’ denotes the location of the selected atom in framework I after being transposed on the reference frame; and (< >) indicates the average square distances covered over residues b. 

Lipinski’s rule of 5 or Ro5 [38] was followed to evaluate any drug-like properties of the 24 phytochemicals of *Artemisia herba-alba*. According to Lipinski, the rule mentions that molecules that are poorly absorbed by the intestinal wall and that are not showing drug-like behavior would present two or more of these characteristics: a molecular weight over than 500, a lipophilicity (log P > 5), hydrogen-bond (HB) donor groups (expressed as the sum of OHs and NHs groups) more than 5, more than 10 HB acceptor groups (expressed as the sum of Os and Ns atoms), and molar refractivity outside a range of 40–130.

## 3. Results and Discussion

### 3.1. Ethnobotanical Aspects of Artemisia herba-alba

SARS-CoV-2 infection and its comorbidities are particularly dangerous for older people who have comorbidities such as diabetes, hypertension, lung, liver, kidney diseases, and cancer [6]. In addition, malaria, tuberculosis, and acquired immune deficiency syndrome (AIDS) can add potential complications to COVID-19 patients in many sub-Saharan African countries [8,9,39]. For example, the similar generic symptoms between COVID-19 and malaria also make immediate diagnosis difficult, causing both diseases to progress. From that viewpoint, the choice of *Artemisia herba-alba* for our present study makes good sense because the plant is quite prolific in northern African countries and contains the current antimalarial drug artemisinin in amounts that are possibly greater than the compound’s current plant source *Artemisia annua* (note that the drug has not been possible to be chemically synthesized) [22,40]. Although one or two papers on the presence of artemisinin in *Artemisia herba-alba* do not make the plant an automatic choice (artemisinin is also present in *Artemisia annua*), the present selection of this plant was based on consideration of other factors as well, such as ethnic uses and pharmacological activities, which were deemed to be suitable for further studies towards alleviating COVID-19 symptoms and comorbidities.

This roadmap is not a comprehensive list of ethnic uses of *Artemisia herba-alba*. Only ethnic reports based on their relevancy to COVID-19 symptoms and comorbidities are presented here. The English name for *Artemisia herba-alba* is wormwood or desert wormwood; in Arabic, it is known as Shih, and in French, it is known as Armoise blanche. In the folk medicine of northeastern Morocco (otherwise known as Oriental Morocco), as well as in Errachidia province in southeastern Morocco, the plant is used to treat arterial hypertension and diabetes; in Tunisian folk medicine, the plant is used for the treatment of bronchitis, diabetes, diarrhea, hypertension, and neuralgia [41,42]. The Bedouins of the Negev desert in Israel use the plant to alleviate stomach disorders [43]. People living in the Middle Atlas Mountains in Morocco use a decoction of fresh leaves and stems of this plant to make herbal tea, which is taken orally 2–3 times a day for a week for the treatment of gastrointestinal disorders [44]. The traditional practitioners, healers, and herbalists of M’sila city in the central part of northern Algeria use the whole plant in decoction, tisane, powder, and in the masticated form for treatment of diabetes, helminthic infections, colonopathy, nausea, abdominal pain, breast pain, and as antispasmodic and carminative [45].

The leaves and flowers of the plant are considered hypoglycemic by people in the Souk Ahras District in Algeria [46]. The leaves of the plant are considered anti-diabetic, antispasmodic, pectoral, and anti-arthritic in the folkloric medicine of Jordan [47]. The decoction of the leaves and flowers of the plant are used for gargling to treat bad breath, gingivitis, toothache, gingival bleeding, mouth ulcers, herpes labialis, and stomatitis by herbalists in the central Middle Atlas (Morocco) [48]. In two Saharan regions of southwest Algeria (Adrar and Bechar), traditional healers administer a decoction of the aerial parts of the plant orally for stomachache and ulcers [49]. In Pakistan, a decoction of the plant is used to treat fever and nervous problems [50]. Taken together, *Artemisia herba-alba*, if the traditionalists are proved correct in clinical trials, by itself can possibly assuage many of the symptoms of COVID-19 as well as serious comorbidities such as malaria, bronchitis, diabetes, diarrhea, hypertension, neuralgia, abdominal pain, and high fever. 

### 3.2. Pharmacological Aspects of Artemisia herba-alba

A recent clinical study with a limited number of patients (*n* = 21) showed that deceased severely ill COVID-19 patients (*n* = 7) displayed higher plasma oxidative stress levels (i.e., higher plasma 4-hydroxynonenal, HNE) than their survived counterparts (*n* = 14) [51]. HNE is a compound produced by lipid peroxidation in cells. Furthermore, their immunohistochemistry data showed increased edema in the alveoli of deceased patients with eosinophilic liquid rich in HNE [52]. Noticeably, the authors of this study reported a low (below the detection limit) total antioxidant capacity of all of the severely ill COVID-19 patients (*n* = 21) reported in their study [52]. Thus, the results from the study indicated that the intake of antioxidant-rich food or dietary materials might help to protect people from oxidative damages caused by COVID-19 or related viral infections. A number of scientific reviews have detailed the pharmacological activities of *Artemisia herba-alba* and its parts, but only reviews pertinent to the alleviation of COVID-19 symptoms and comorbidities will be presented and discussed. One review mentions the activities as being antioxidant, anti-venom, anti-fungal, nematocidal, antibacterial, antispasmodic, anthelmintic, anti-leishmanial, anti-neurological disorders (Alzheimer’s disease, epilepsy, and depression), and hypoglycemic [31]. A second review mentions the pharmacological activities of the plant as anti-diabetic, anti-hypertensive, antioxidant, anti-fungal, neurological, antimalarial, antispasmodic, immunomodulatory, and anti-mycoplasmal [53]. The aqueous extract of the aerial parts of the plant was reportedly active against *Escherichia coli*, *Serratia marcescens* W225, *Enterobacter cloacae*, *Shigella dysenteriae*, *Proteus vulgaris*, *Bacillus subtilis*, and *Staphylococcus aureus* [54]. In a clinical trial with 15 diabetic patients, 14 patients showed the lowering of elevated blood sugar and the good remission of diabetic symptoms following the administration of the plant extract [55]. A 70% ethanol extract of the dried plant has been shown to inhibit α-amylase activity in vitro and lower elevated plasma glucose levels in alloxan-diabetic rats in vivo [56]. Overall, a conclusion can be arrived at regarding the results of pharmacological activity studies on the plant, which corroborate well with the plant’s ethnic uses, and a number of the various ailments that ethnic uses and pharmacological activities correspond to match with the comorbidities or symptoms generally observed in COVID-19 patients. If this conclusion is correct, then that justifies further in silico studies on the phytochemicals of *Artemisia herba-alba* against the SARS-CoV-2 virus itself.

### 3.3. Molecular Docking Studies of Artemisia herba-alba Phytochemicals with SARS-CoV-2 Mpro

**Figure 1:**
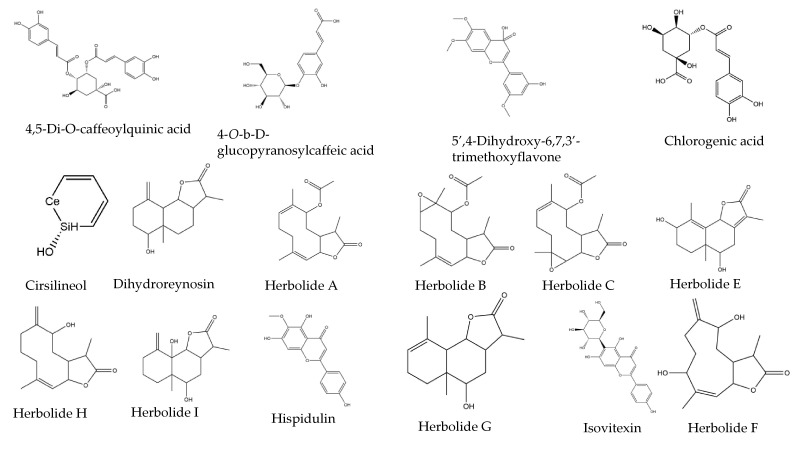

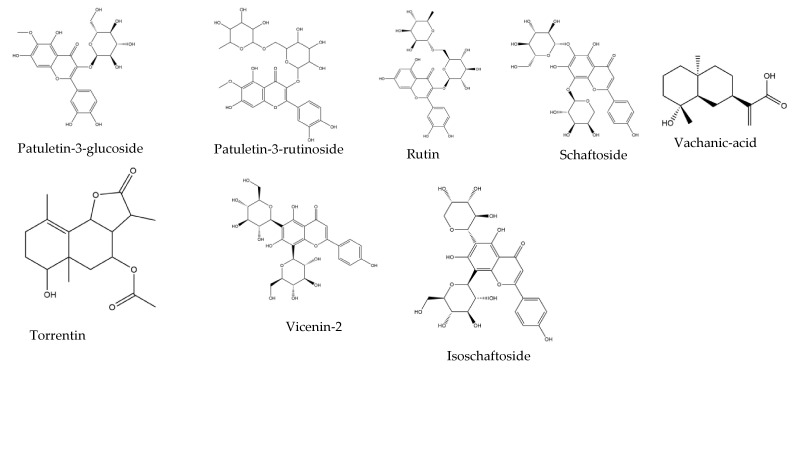
Structures of various phytochemicals of *Artemisia herba-alba*.

The predicted binding energies of the 24 *Artemisia herba-alba* phytochemicals to SARS-CoV-2 Mpro (chymotrypsin-like main protease) are shown in Table 1, and their structures are shown in Figure 1. These phytochemicals were randomly chosen since it was not possible to perform binding studies with all of the phytochemicals of the plant.

Phytochemicals showing predicted high binding affinities to Mpro (a high binding affinity that is a low binding energy is taken as equal to or less than −7.5 kcal/mol) included 4,5-di-*O*-caffeoylquinic acid (−8.5), chlorogenic acid (−7.5), hispidulin (−7.8), isovitexin (−7.8), patuletin-3-glucoside (−8.0), patuletin-3-rutinoside (−7.9), rutin (−8.8), schaftoside (−8.3), and vicenin-2 (−7.9). The putative SARS-CoV-2 Mpro inhibitors lopinavir and nelfinavir [57] showed predicted binding energies to Mpro at −8.2 and −8.1, respectively. The predicted binding energies show that at least 3 of 24 phytotochemicals, namely 4,5-di-*O*-caffeoylquinic acid, rutin, and schaftoside possessed higher binding energies than the inhibitors.

Incidentally, chlorogenic acid, dicaffeoyl quinic acids, and hispidulin have been reported to inhibit SARS-CoV [58]; H1N1 (influenza A virus), Coxsackievirus B3, and herpes simplex virus 1 [59]; and Respiratory Syncytial Virus [60], respectively. *Desmodium canadense extract* containing isovitexin reportedly showed inhibitory activity against avian infectious bronchitis virus (IBV) [61]. Other in silico studies have shown the potential of rutin as an inhibitor of SARS-CoV-2 [62]. The inhibition of a different virus is not tantamount to inhibiting SARS-CoV-2, but, in a predictive sense, it may be considered to have a higher probability of acting against the COVID-19 virus [63], more so if there is a high degree of sequence homology and other similarities between the two viruses.

**Figure 2:**
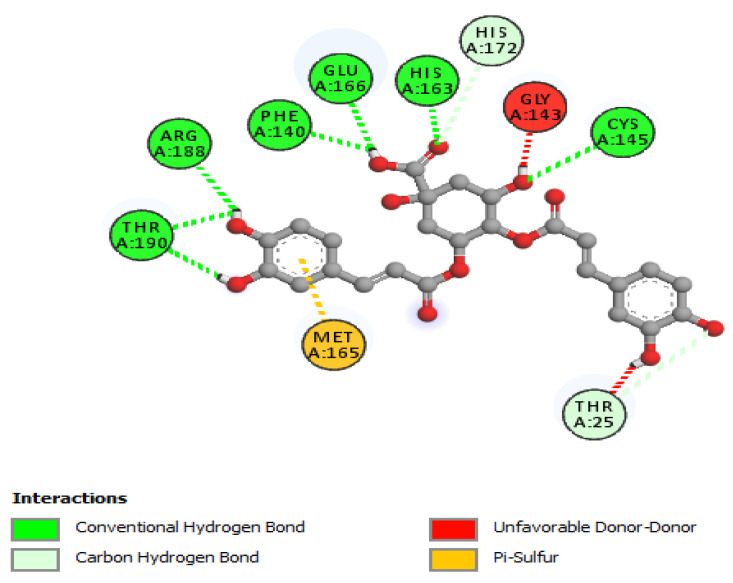
2D interactions of 4,5-di-*O*-caffeoylquinic acid with Mpro.

**Figure 3:**
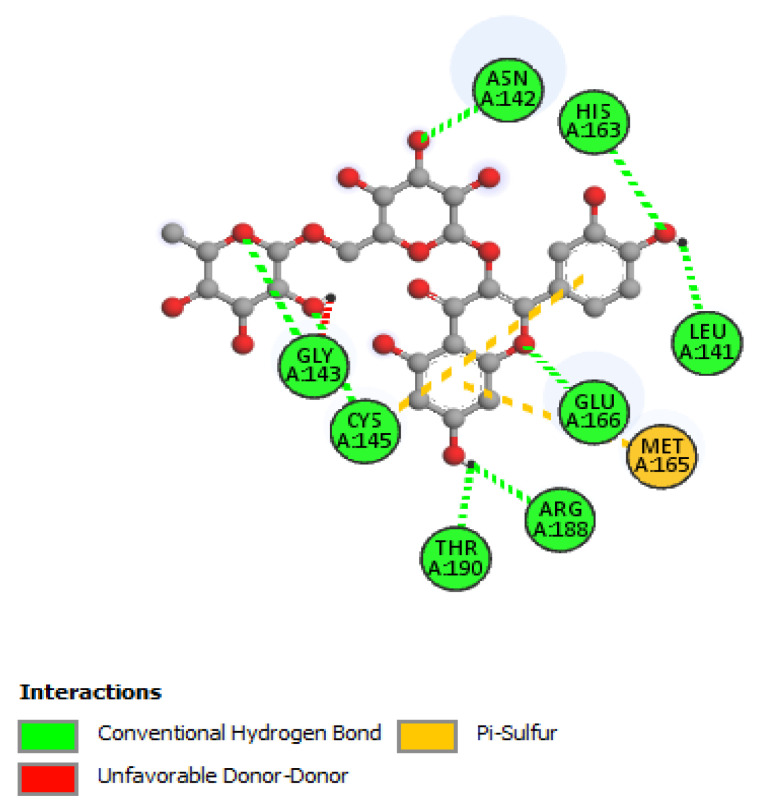
2D interactions of rutin with Mpro.

**Figure 4:**
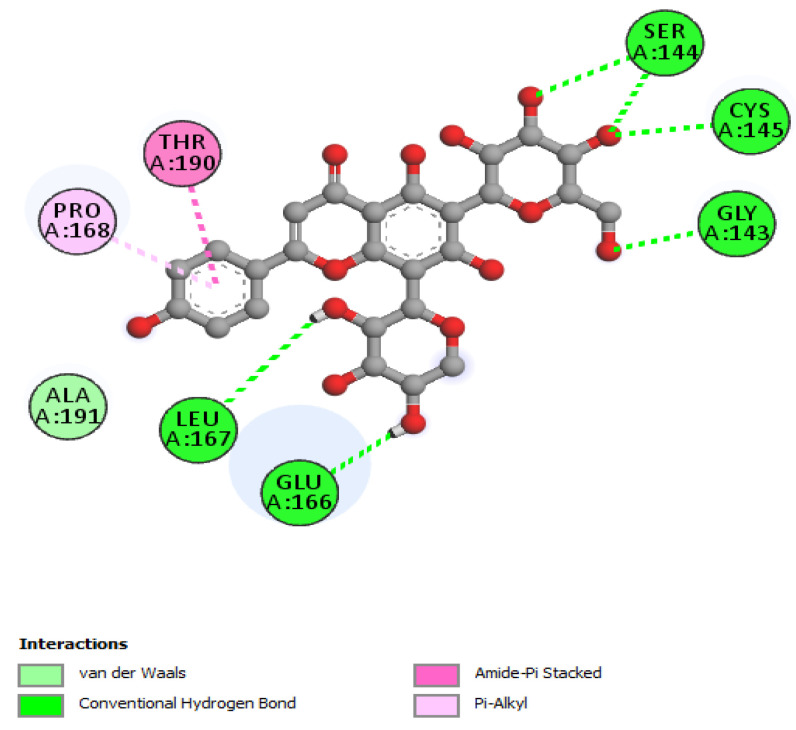
2D interactions of schaftoside with Mpro.

**Figure 5:**
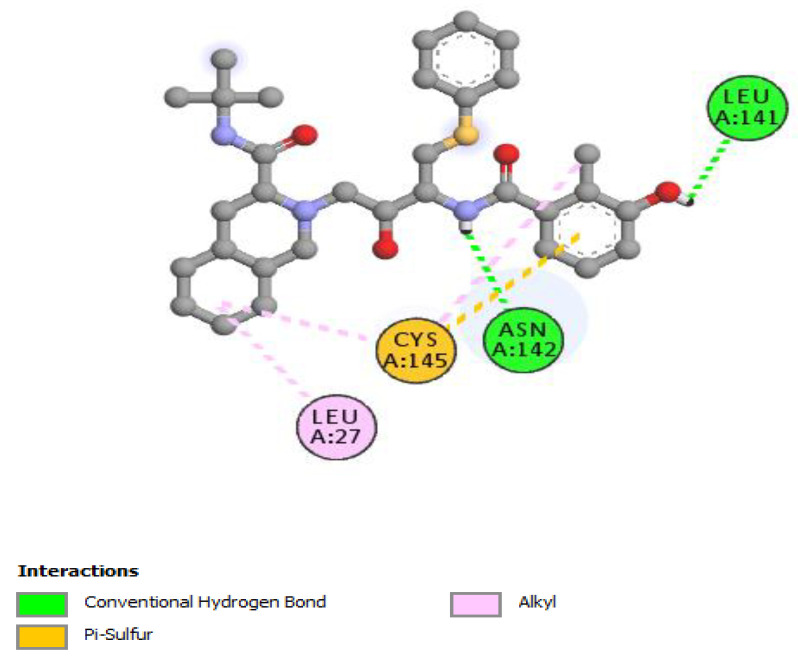
2D interactions of nelfinavir with Mpro.

**Figure 6:**
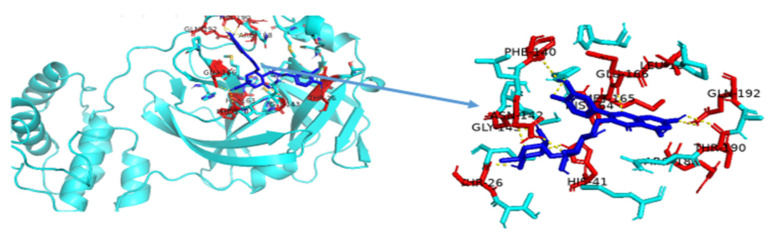
Binding of rutin to Mpro. Cyan-colored cartoon structure is Mpro and blue-colored stick structure is the ligand, rutin. Additionally, red-colored amino acid residues are involved in polar interactions with rutin.

The non-bonding interactions of the *Artemisia herba-alba* phytochemicals (top three compounds with highest binding affinities to Mpro amino acid residues) are shown in Table 2. These three compounds are rutin, 4,5-di-*O*-caffeoylquinic acid, and schaftoside, with predicted binding energies, respectively, of −8.8, −8.5, and −8.3 kcal/mol. Mpro is 306 amino acids long; it has been reported to contain a catalytic dyad comprising of His41 and Cys145. The protease exists as a dimer, with the monomeric form being essentially inactive [64,65]. Each monomer contains three domains; domain 1 comprises amino acid residues 8–101, domain 2 contains amino acid residues 102–184, and domain 3 contains residues 201–306. A loop region (amino acid residues 185–200) connects domain 2 with domain 3. The substrate-binding site location is within a cleft formed between domains 1 and 2 [66]. The interactions of the three phytochemicals, as shown in Table 2, demonstrate that all three phytochemicals bind to one or both amino acids of the catalytic dyad and mainly form strong interactions with the amino acid residues of domain 2. The same holds true for other phytochemicals from *Artemisia herba-alba,* which show predicted high binding energies to Mpro (≥7.5 kcal/mol); with the exception of vicenin-2 and isovitexin, several other phytochemicals that were evaluated, such as chlorogenic acid, hispidulin, patuletin-3-glucoside, and patuletin-3-rutinoside, demonstrated interactions with one or both of the catalytic dyad amino acid residues His41 and Cys145 (data not shown). Overall, the results from Table 1, Table 2 and Table 3, as well as those shown Figure 2, Figure 3, Figure 4, Figure 5 and Figure 6 and other Figures (data not shown) suggest that phytochemicals from *Artemisia herba-alba* have a strong probability of binding to SARS-CoV-2 Mpro, inhibiting the protease with the consequential blockage of viral replication (compare binding with nelfinavir to Mpro shown in Figure 5). It is also possible that when administered as a crude extract, as is the case in herbal medicines for a number of COVID-19 comorbidities, the phytochemicals may produce a synergistic effect with regard to binding to and inhibiting Mpro. 

The physicochemical properties of ten of the predicted high energy binding phytochemicals to Mpro are given in Table 3. As per Lipinski’s rule of five, chlorogenic acid, hispidulin, isovitexin, and patuletin-3-glucoside all have two or fewer violations and can serve as drug candidates against SARS-CoV-2 (the known Mpro inhibitors lopinavir and nelfinavir had two and one violations, respectively). However, although rutin, schaftoside, and 4,5-di-*O*-caffeoylquinic acid have three violations each, they also have predicted high binding energies to one or both amino acids of the catalytic dyad of Mpro and as such possess high possibilities as therapeutics. Lipinski’s rule essentially indicates the bioavailability of a given compound; the lesser the violations, the more bioavailable the compound is. From that viewpoint, rutin, schaftoside, and 4,5-di-*O*-caffeoylquinic acid would be less bioavailable and would be of lesser value as oral drugs. On the other hand, a number of commonly used oral conventional drugs violate Lipinski’s rules but still work effectively. 

Rutin has previously been shown to have a high affinity for binding at the substrate-binding site of Mpro and to interact with the catalytic dyad amino acid residues His41 and Cys145 [67,68]. The amino acid residues of Mpro reported in the substrate-binding site include Thr25, Thr26, Cys44, Thr45, Ser46, Met49, Tyr54, Asn119, Asn142, Cys145, His164, Met165, Glu166, Val186, Asp187, Arg188, Thr190, Ala191, Gln189, and Gln192 [69]. In the present study, which involved molecular docking, we found that rutin interacts with the amino acid residues of Mpro, including Thr26, Asn 142, Met165, Glu166, and Thr190 along with His41 and Cys145, thus forming a strong interaction with the protease overall (see also Table 2 and Figure 6). Interestingly, a review paper on the antiviral activities of plant polyphenol compounds list rutin to have demonstrated antiviral activity against rabies, parainfluenza, potato, influenza, and dengue viruses [70]. 

Antiviral activities have been reported for other phytochemicals of *Artemisia herba-alba*. The antiviral activity of the Geopropolis extract of *Scaptotrigona affinis* postica (Latreille, 1807), (Hymenoptera, Apidae, Meliponini) against the Rubella virus has been attributed to its apigenin derivatives, schaftoside and vicenin-2 [71]. Schaftoside has also been reported as a potential inhibitor of dengue virus NS2B-NS3 protease [72]. In a recent review, Di-caffeoylquinic acids have been mentioned to be active against influenza A virus (H1N1), Coxsackiue virus B3 (CVB3), and herpes simplex virus-1 (HSV-1) [73]. As such, the compounds rutin, schaftoside, and 4,5-di-*O*-caffeoylquinic acid seem to be active against a number of viruses and may also show inhibitory activity against SARS-CoV-2. The same review [74] further mentioned cirsilineol to be active against the Orthomyxovirus and Paramyxovirus, chlorogenic acid against the Severe Acute Respiratory Syndrome Coronavirus (SARS-CoV), and hispidulin against the Respiratory Syncytial Virus (RSV). Patuletin 3-*O*-beta-d-glucopyranoside reportedly possesses antioxidant properties, which can be useful against COVID-19 [75]. Notably, all of these compounds are present as phytochemicals in *Artemisia herba-alba*.

The potential for a given compound or a given plant extract (containing a mixture of phytochemicals) for the simultaneous treatment of COVID-19 and comorbidities opens up a unique opportunity for treatment and saving lives. *Artemisia herba-alba*, although discussed extensively in this manuscript, is not the only plant in this regard. Another example can be found in *Tinospora cordifolia*, a plant found extensively in the Indian sub-continent, that possesses the alkaloid berberine. In clinical trials, berberine has shown its effectiveness as a potent oral hypoglycemic agent with additional benefits on lipid metabolism. Berberine is also known to inhibit the replication of the herpes simplex virus (HSV), human cytomegalovirus (HCMV), human papillomavirus (HPV), and human immunodeficiency virus (HIV) and has the potential to be effective against SARS-CoV-2. In silico studies have also revealed that berberine has the ability to bind and inhibit Mpro [76]. Diabetes was estimated to affect around 463 million persons in the world in 2019 [77], and 17.4% of COVID-19 patients have diabetes as a comorbidity [7]. Berberine has been reported to show anti-proliferative and cytotoxic effects in a number of cell lines, including human cancer HeLa and murine leukemia L1210 cells; MCF-7 human breast cancer cells, human epidermoid carcinoma A431 cells, human gastric carcinoma SNU-5 cells, androgen insensitive (DU145 and PC-3), and androgen-sensitive (LNCaP) prostate cancer cells; HepG2 cells, human promonocytic U937 cells, HCT-116 human colorectal cancer cells, mouse K1735-M2, and human WM793 melanoma cells; and human glioblastoma T98G cells, reviewed in [78], and so can be of use in COVID-19 patients with various cancer types and/or diabetes. 

### 3.4. Molecular Dynamic Simulation of Artemisia herba-alba Phytochemicals with High Affinity Binding with SARS-CoV-2 Mpro 

Molecular dynamics simulation (MD simulation) is performed in computer-aided drug discovery to understand the stability and intermolecular interaction of a protein–ligand complex on a real-time basis. This technique can also determine the conformational change of a complicated system when it is exposed to an artificial environment. A 100 ns MD simulation of the protein in association with the specific ligand was conducted in this work in order to better understand the conformational changes of the protein in the complex. Analysis of intermolecular behavior was first carried out using the 100 ns terminal snapshots of the MDS trajectories.

The average change in the root means square deviation (RMSD) of the protein–ligand interaction is perfectly acceptable and occurs within a range of 1–3 Å. If the RMSD value is greater than 1–3, it indicates that the protein structure has undergone a significant conformational change. Accordingly, to determine the conformational change of the desired protein in the complex with the three ligand compounds including 4,5-di-*O*-Caffeoylquinic acid (CID-6474309), rutin (CID-5280805), and schaftoside (CID-442658), a 100 ns MD simulation was performed, and the corresponding RMSD value was calculated. 

**Figure 7:**
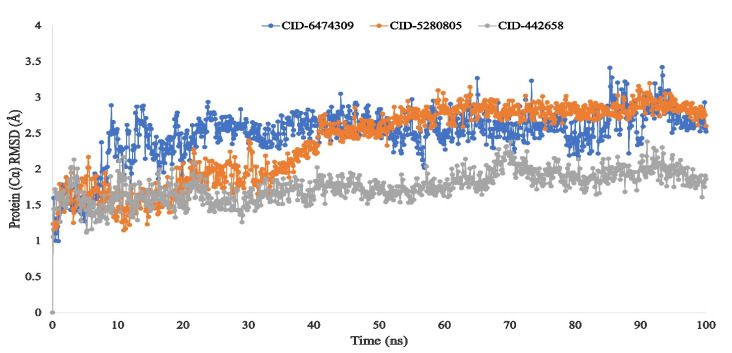
The RMSD values for the SARS-CoV-2 Mpro protein complexed with the three ligand compounds recovered from the complex system’s Cα atoms. The selected three ligands compound 4,5-di-*O*-Caffeoylquinic acid (CID-6474309), rutin (CID-5280805), and schaftoside (CID-442658) in association with the selected protein are depicted by green, orange, and grey, respectively.

For the ligand compound schaftoside (CID-442658), the average root mean square deviation (RMSD) was between 1.5–2.3 Å. Another two ligand compounds, 4,5-di-*O*-Caffeoylquinic acid (CID-6474309) and rutin (CID-5280805), also possessed a potent RMSD value in an associated range of 1.5–3 Å. The change in the value of the compound exhibits very little fluctuation that is less than the acceptable range, indicating the conformational stability of the protein–ligand complex structure depicted in Figure 7. 

**Figure 8:**
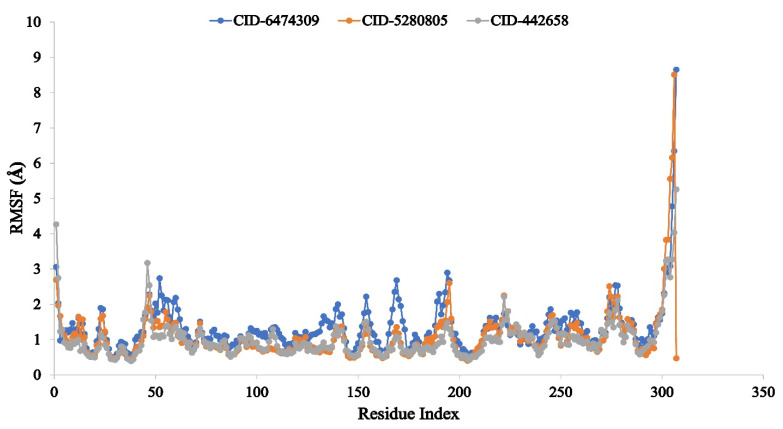
The RMSF values were determined for the protein Cα atoms in the docked protein–ligand complexes. The selected three ligand compounds 4,5-di-*O*-Caffeoylquinic acid (CID-6474309), rutin (CID-5280805), and schaftoside (CID-442658) in association with the selected protein are depicted by green, orange, and grey, respectively.

The Root Mean Square Fluctuation (RMSF) can assist in characterizing and determining the local changes that occur within the protein chain when selected ligand compounds that interact with certain residues. Consequently, the RMSF values of the compounds 4,5-di-*O*-Caffeoylquinic acid (CID-6474309), rutin (CID-5280805), and schaftoside (CID-442658) in complex with the COVID-19 Mpro protein were calculated in order to analyze the alteration in protein structural flexibility caused by the attachment of the selected ligand compounds to a specific residual position, as shown in Figure 8.

It was discovered that the most rigid secondary structural elements, such as alpha-helices and beta-strands, had a minimum observation rate in the range of 5 to 290 amino acids residues. Because of the existence of the N- and C-terminal domains, the majority of the fluctuation is observed at the beginning and end of the protein. As a result, it can be determined that in the simulation environment, the displacement of an individual atom has a low fluctuation probability for the three ligand compounds studied.

The radius of gyration (Rg) of a protein–ligand interaction system can be characterized as the arrangement of its atoms across its axis. When predicting a macromolecule’s structural functionality, the calculation of Rg is one of the most essential indicators to look for because it reveals changes in complex compactness over time.

**Figure 9:**
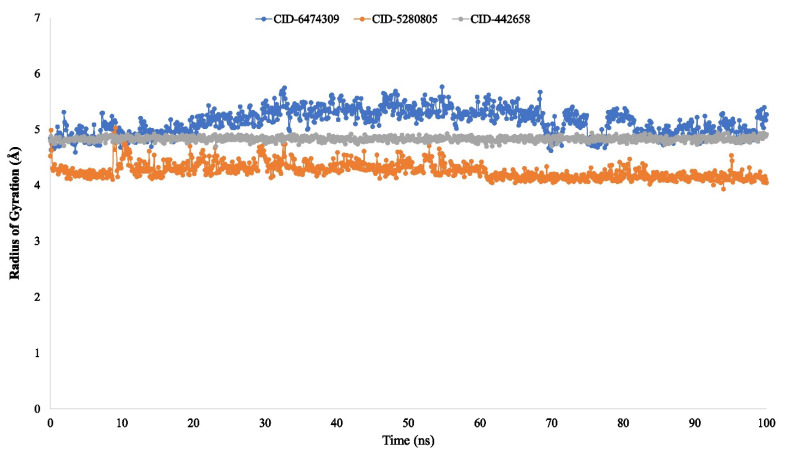
The 100 ns simulation was performed to determine the radius of gyration (Rg) of the protein-ligand interaction. The selected three ligands compound 4,5-di-*O*-Caffeoylquinic acid (CID-6474309), rutin (CID-5280805), and schaftoside (CID-442658) in association with the selected protein are depicted by green, orange, and grey, respectively.

As a result, the stability of 4,5-di-*O*-Caffeoylquinic acid (CID-6474309), rutin (CID-5280805), and schaftoside (CID-442658) in association with the target protein was also investigated in terms of Rg over a 100-ns simulation duration, as demonstrated in Figure 9. The average Rg values for the compounds 4,5-di-*O*-Caffeoylquinic acid (CID-6474309), rutin (CID-5280805), and schaftoside (CID-442658) were determined to be 5.4, 4.4, and 4.8, respectively, showing that the protein’s binding site does not undergo significant structural alterations upon binding the selected ligand compounds.

The quantity of solvent-accessible surface area (SASA) on biological macromolecules determines their organization and activities. In most situations, amino acid residues on a protein’s surface function as active sites and/or interact with other molecules and ligands, which improves the understanding of a molecule’s solvent-like behavior (hydrophilic or hydrophobic) as well as the protein–ligand interaction components.

**Figure 10:**
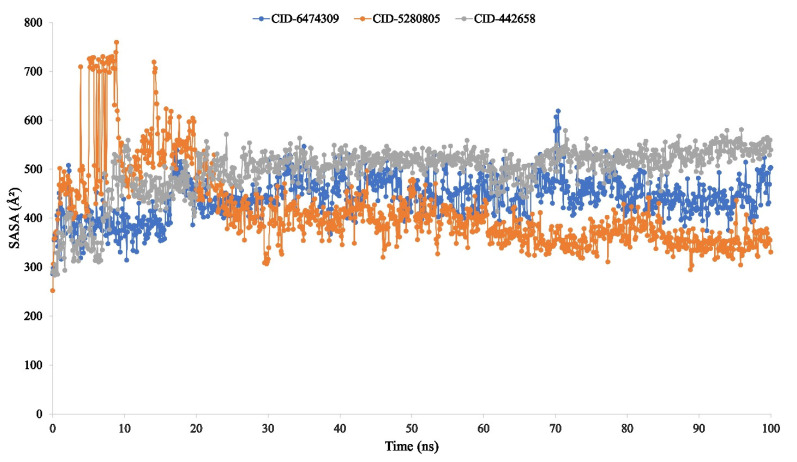
The solvent-accessible surface area (SASA) of the protein–ligand interaction compounds was calculated from the 100 ns simulation interaction diagram. The selected three ligands compound 4,5-di-*O*-Caffeoylquinic acid (CID-6474309), rutin (CID-5280805), and schaftoside (CID-442658) in association with the selected protein are depicted by green, orange, and grey, respectively.

As a result, the SASA value for the protein complexed with the three ligand compounds 4,5-di-*O*-Caffeoylquinic acid (CID-6474309), rutin (CID-5280805), and schaftoside (CID-442658) was determined and plotted in Figure 10. The SASA value determined for the compound structure was on average between 250 and 700 A2, indicating that an amino acid residue was exposed to a high amount of the selected ligand compounds in the complex systems.

**Figure 11:**
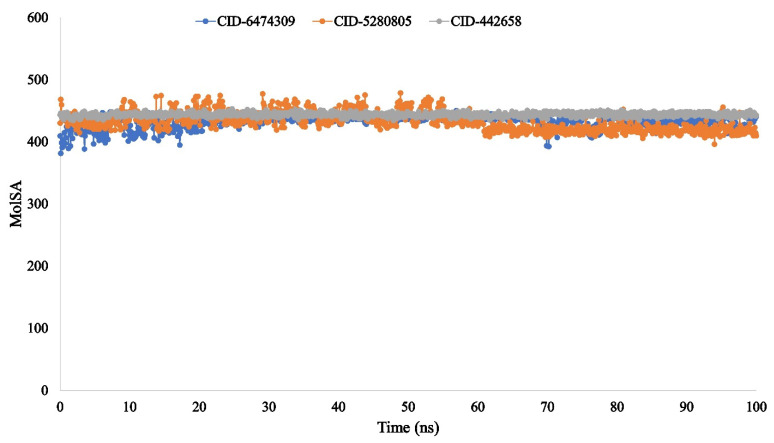
The molecular surface area (MolSA) of the protein–ligand interaction compounds was calculated from the 100 ns simulation interaction diagram. The selected three ligands compound 4,5-di-*O*-Caffeoylquinic acid (CID-6474309), rutin (CID-5280805), and schaftoside (CID-442658) in association with the selected protein are depicted by green, orange, and grey, respectively.

**Figure 12:**
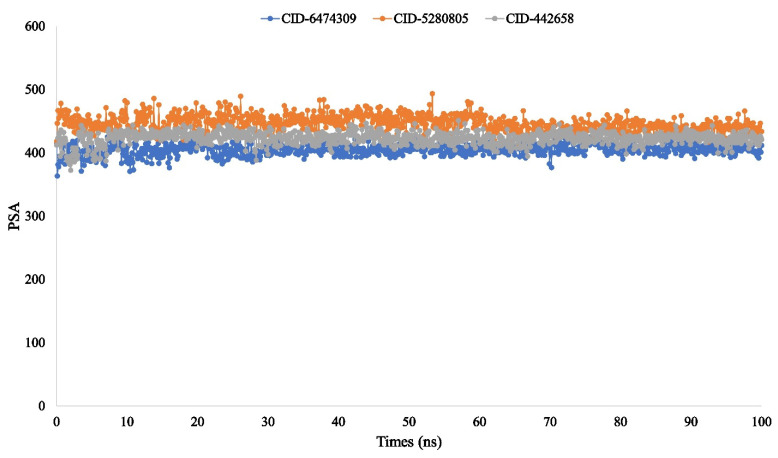
The polar surface area (PSA) of the protein–ligand interaction compounds was calculated from the 100 ns simulation interaction diagram. The selected three ligands compound 4,5-di-*O*-Caffeoylquinic acid (CID-6474309), rutin (CID-5280805), and schaftoside (CID-442658) in association with the selected protein are depicted by green, orange, and grey, respectively.

The molecular surface area (MolSA) is equivalent to a van der Waals surface area, which is calculated with a 1.4 Å probe radius. In our in silico study, all of the ligand compounds, 4,5-di-*O*-Caffeoylquinic acid (CID-6474309), rutin (CID-5280805), and schaftoside (CID-442658), possessed the standard van der Waals surface area (Figure 11). Additionally, the polar surface area (PSA) in a molecule is only contributed to by oxygen and nitrogen atoms. Here, all the ligand compounds 4,5-di-*O*-Caffeoylquinic acid (CID-6474309), rutin (CID-5280805), and schaftoside (CID-442658) exhibited a strong PSA value with the targeted protein (Figure 12).

An analysis of Intramolecular Bonds was conducted through the simulation interactions diagram (SID), the complex configuration of a protein with the specified ligands and their intermolecular interactions were analyzed for a 100 ns simulation time.

**Figure 13:**
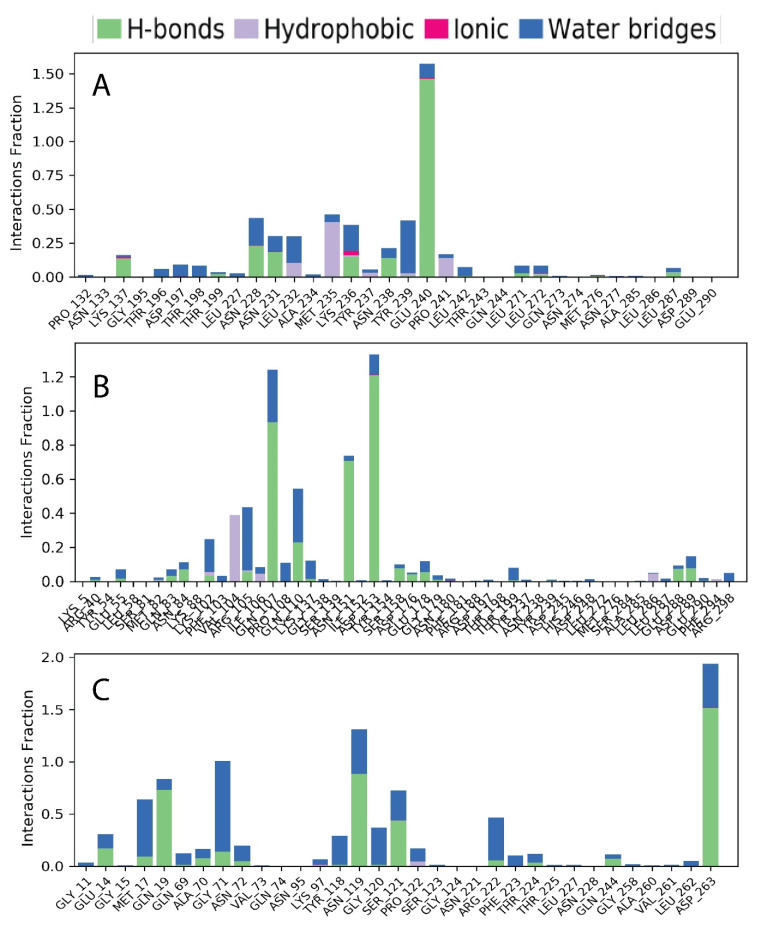
The stacked bar charts illustrate the interactions between proteins and ligands discovered during the 100 ns simulation. The interaction of the three ligand compounds (**A**) 4,5-di-*O*-Caffeoylquinic acid (CID-6474309), (**B**) rutin (CID-5280805), and (**C**) schaftoside (CID-442658) in association with the selected protein is illustrated in this section.

The interactions between the protein and the selected ligand compounds were influenced by a number of different parameters, such as the noncovalent bond (hydrophobic bond), the hydrogen bond, the ionic bond, and the water bridge bond in which (Figure 13), which are analyzed and represented as (A) 4,5-di-*O*-Caffeoylquinic acid (CID-6474309), (B) rutin (CID-5280805), and (C) schaftoside (CID-442658). Throughout the 100 ns simulation time, it was discovered that all of the compounds formed multiple connections via hydrogen, hydrophobic, ionic, and water bridge bonding and maintained these contacts until the simulation ended, assisting in the formation of stable binding with the desired protein. 

The stability of a protein in a complex with ligands can be confirmed via molecular dynamic simulation [79,80]. It can also access the stiffness in the stability of the protein–ligand complexes in a particular environment such as the human body [36]. A compound’s maximum stability is represented by its RMSD, but its mean fluctuations, which define the protein–ligand complex compactness, are determined by its RSMF values [81]. 

The RMSD of the system was calculated using the actions of the protein–ligand complex, confirming the protein’s minimum fluctuation. The RMSF value was used to access the protein fluctuation, which also demonstrated lower fluctuation, indicating the stability of the compound to the target protein. In our in silico study, the ligand compounds 4,5-di-*O*-Caffeoylquinic acid (CID-6474309), rutin (CID-5280805), and schaftoside (CID-442658) have been demonstrated strong RMSD and RMSF values in association with the selected SARS-CoV-2 Mpro protein.

The radius of gyration (Rg) estimated the protein’s center of mass from the C and N terminals and analyzed protein folding properties [82]. The lower Rg value indicates great compactness, whereas the higher Rg value indicates the disassociation of the compounds from the protein [83]. The higher the SASA value, the less stable the structure, and the lower the SASA value, the more closely compressed the complex of water molecules and amino acid residues [84,85]. The Rg, SASA, MolSA, and PSA values of the three ligand compounds that studied were determined to be at their optimum level in the research study. 

### 3.5. Reported Effect(s) of High Affinity Mpro Binding Phytochemicals of Artemisia herba-alba on Selected Comorbidities

About a quarter of allopathic drugs have been discovered from medicinal plants following ethnomedicinal leads prior to isolating the active ingredient [86]. What we are proposing is that under pandemic-like situations involving new infectives and where the rapid discovery of therapeutics becomes a prime necessity, a judicious combination of ethnic use, phytochemical, and pharmacological reports in combination with in silico studies can lay the basic framework of identifying phytochemicals for further analysis and clinical trials. Plants are being proposed because they are readily available, can form a very diverse source of bioactive compounds, and are studied with comparative ease.

There are a substantial number of examples of mono-herbal or poly-herbal formulations, which are in use against various COVID-19 comorbidities or that are even undergoing trial against COVID-19. However, the use of a single plant or its phytochemical(s) or formulation for the dual treatment of COVID-19 and its comorbidities appears to have not been investigated with the attention that the subject deserves. Yet, the use of a single plant avoids the need to find a number of plants together in the same season and preferably within reasonable collecting distances of one another. Traditional Chinese medicines (TCMs) such as ‘Yu Ping Feng San’ (containing *Astragali radix*, *Astragalus membranaceus*, *Atractylodes macrocephala*, and *Saposhnikoviae radix*), which are used traditionally for ‘tonifying qi’ to protect from external pathogens, or ‘Shuang Huang Lian’ (containing *Lonicera japonica*, *Scutellaria baicalensis*, and *Forythia suspensa*), used traditionally to ‘clear heat and detoxify, remove lung hotness’, among other TCMs, are potential candidates for SARS-CoV-2 treatment; clinical trials are underway with a number of TCMs such as Tan Re Qing, Kang Bing Du, and Jing Yin for the treatment of COVID-19 pneumonia, to name only a few. Further details on these TCMs can be found in [87]. However, to our knowledge, any study dealing simultaneously with the treatment of COVID-19 with a single phytochemical or formulation or extract from one given plant species is still lacking despite its enormous promise, as shown in the present study. Questions such as how to administer crude extracts effectively may be raised, but this can easily be solved by preparing and using nanoparticles [88,89,90]. 

There has been a substantial number of in silico studies of the possible inhibition of SARS-CoV-2 through the binding of its protein components with various groups of phytochemicals or through inhibiting binding to its receptor hACE2. However, any effective drug has not emerged, at least thus far, from these in silico studies. In our opinion, this calls for more studies to determine the possible cause(s) of these ‘failures’ and to devise how these shortcomings may be overcome. Furthermore, it needs to be pointed out that more studies in the future should not only include experiments on COVID-19 and phytochemical(s) from this plant, but also scientific studies with phytochemicals of the plant against comorbidities, such as malaria and diabetes. 

## 4. Conclusions

COVID-19 is caused by the coronavirus SARS-CoV-2 and is the cause of the recent pandemic. Thus far, a drug against COVID-19 has yet to be discovered. Several vaccines have been approved on an ‘emergency approval’ basis, but without the knowledge of their long-term effects. COVID-19 is a complicated disease that can cause fatalities, with 84.1% of the patients with comorbidities dying. Elderly persons with one or more comorbidity have an increased chance of being affected by the virus; once infected, COVID-19 can be the cause of one or more comorbidity, including multi-organ failure. In this manuscript, we show how an informed use of a single plant and its phytochemicals, extracts, and formulations can be used to target both COVID-19 and one or more comorbidity simultaneously. This approach can be affordable (unlike COVID-19 treatment) and can serve the dual or even multi-purpose of getting COVID-19 and comorbidity/comorbidities under control. We show that various phytochemicals of *Artemisia herba-alba* can be useful against COVID-19 (in silico) and diabetes, malaria, and other diseases (ethnic uses thus far, but clinical trials can be started at an early date). We also provide evidence from the available scientific literature that other phytochemicals such as berberine, a phytochemical present in the plant *Tinospora cordifolia*, has been shown to be effective against diabetes and cancer (in vivo studies) and SARS-CoV-2 (in silico). Cumulatively, we demonstrate that through proper selection of medicinal plants and the phytochemicals present therein, COVID-19 and comorbidities may be treated, or at the very least, the symptoms associated with it may be alleviated, possibly leading to decreased fatalities. In this current in silico study, these three selected ligand compounds, namely 4,5-di-*O*-Caffeoylquinic acid, rutin, and schaftoside have been shown to demonstrate strong stability with the SARS-CoV-2 Mpro protein complex in molecular dynamic simulation study in a 100 ns time interval. In the RMSD and RMSF calculations, these compounds possessed lower fluctuation with the protein complex. Moreover, in SASA, Rg, MolSA, and PSA validation, these three compounds showed potential results. In contrast, there is no sufficient clinical research evidence based on these compounds for the treatment of SARS-CoV-2. Therefore, after the validation of anti-SARS-CoV-2 activity in in vitro and in vivo research models, these potential bioactive phyto-compounds can prove to be an alternative therapeutic option for SARS-CoV-2 treatment. To conclude, this study connects how the ethnic usages of a given medicinal plant coupled with pharmacological activity reports and the in silico screening of its phytochemicals can lead to the better management of COVID-19, which is extremely important for dealing with COVID-19 and its associated comorbidities.

## Figures and Tables

**Table 1:** Predicted binding energy in molecular docking studies of some *Artemisia herba-alba* phytochemicals to Mpro of SARS-CoV-2.

Phytochemical	Binding Energy (ΔG = kcal/mol) to Mpro
4,5-di-*O*-Caffeoylquinic acid	−8.5
4-*O*-b-D-glucopyranosylcaffeic acid	−6.8
5′4-Dihydroxy-6,7,3′-trimethoxyflavone	−7.2
Chlorogenic acid	−7.5
Cirsilineol	−7.3
Dihydroreynosin	−6.7
Herbolide A	−7.0
Herbolide B	−6.2
Herbolide C	−6.5
Herbolide E	−6.5
Herbolide F	−6.4
Herbolide G	−7.1
Herbolide H	−7.2
Herbolide I	−6.9
Hispidulin	−7.8
Isoschaftoside	−7.3
Isovitexin	−7.8
Patuletin-3-glucoside	−8.0
Patuletin-3-rutinoside	−7.9
Rutin	−8.8
Schaftoside	−8.3
Torrentin	−6.3
Vachanic_acid	−6.9
Vicenin-2	−7.9
Lopinavir	−8.2
Nelfinavir	−8.1

**Table 2:** Non-bonding interactions of the top three predicted high binding energy phytochemicals of *Artemisia herba-alba* to Mpro of SARS-CoV-2. (CH = conventional hydrogen bond; C = carbon hydrogen bond).

Rutin			
Interacting Residue	Distance	Category	Type
His41	2.33	Hydrogen Bond	CH
Asn142	2.28	Hydrogen Bond	CH
Gly143	2.27	Hydrogen Bond	CH
Cys145	2.99	Hydrogen Bond	CH
Glu166	2.27	Hydrogen Bond	CH
Phe140	2.85	Hydrogen Bond	CH
Thr190	2.60	Hydrogen Bond	CH
Thr26	1.90	Hydrogen Bond	CH
Cys145	4.97	Other	Pi-Sulfur
Met165	5.12	Other	Pi-Sulfur
**4,5-di-*O*-Caffeoylquinic acid**			
Cys145	2.75	Hydrogen Bond	CH
His163	2.01	Hydrogen Bond	CH
Thr190	2.02	Hydrogen Bond	CH
Arg188	2.43	Hydrogen Bond	CH
Thr190	2.32	Hydrogen Bond	CH
Phe140	2.38	Hydrogen Bond	CH
Glu166	2.25	Hydrogen Bond	CH
Thr25	3.30	Hydrogen Bond	C
His172	3.54	Hydrogen Bond	C
Met165	4.88	Other	Pi-Sulfur
**Schaftoside**			
Gly143	2.18	Hydrogen Bond	CH
Ser144	2.54	Hydrogen Bond	CH
Ser144	2.71	Hydrogen Bond	CH
Cys145	2.73	Hydrogen Bond	CH
Glu166	2.270	Hydrogen Bond	CH
Leu167	3.00	Hydrogen Bond	CH
Thr190; Ala191	3.99	Hydrophobic	Amide-Pi Stacked
Pro168	5.08	Hydrophobic	Pi-Alkyl
**Lopinavir**			
Gln110	2.12	Hydrogen Bond	Conventional Hydrogen Bond
Asp153	2.42	Hydrogen Bond	Conventional Hydrogen Bond
Ile249	3.44	Hydrophobic	Pi-Sigma
Phe294	3.73	Hydrophobic	Pi-Pi Stacked
Pro252	3.96	Hydrophobic	Alkyl
Val104	3.78	Hydrophobic	Alkyl
Phe294	5.19	Hydrophobic	Pi-Alkyl
Pro252	5.39	Hydrophobic	Pi-Alkyl
Val297	5.10	Hydrophobic	Pi-Alkyl
Val202	5.14	Hydrophobic	Pi-Alkyl
**Nelfinavir**			
Asn142	2.25	Hydrogen Bond	Conventional Hydrogen Bond
Leu141	2.88	Hydrogen Bond	Conventional Hydrogen Bond
Cys145	5.90	Other	Pi-Sulfur
Leu27	5.04	Hydrophobic	Alkyl
Cys145	4.98	Hydrophobic	Alkyl
Cys145	4.51	Hydrophobic	Alkyl

**Table 3:** Physico-chemical properties of selected compounds *Artemisia herba-alba*.

Compound Name	Molecular Weight	Number of H-Bond Acceptors	Number of H-Bond Donors	Log P	Molar Refractivity	Number of Violation
4,5-di-*O*-Caffeoylquinic acid	516.45	12	7	1.25	126.90	3
Chlorogenic acid	354.31	9	6	0.96	83.50	1
Hispidulin	300.26	6	3	2.27	80.48	0
Isoschaftoside	564.49	14	10	1.41	133.26	3
Isovitexin	432.38	10	7	1.94	106.51	1
Patuletin-3-glucoside	494.40	13	8	2.45	116.65	2
Patuletin-3-rutinoside	640.50	17	10	1.61	147.87	3
Rutin	610.52	16	10	2.43	141.38	3
Schaftoside	564.49	14	10	1.63	133.26	3
Vicenin-2	594.52	15	11	1.27	139.23	3
Lopinavir	628.80	5	4	3.44	187.92	2
Nelfinavir	567.78	5	4	3.87	166.17	1

## Data Availability

Not applicable.

## Sample Availability

Not available.
